# Collagen-targeted tracers for molecular imaging of atrial fibrosis and sensitive detection of atrial fibrillation

**DOI:** 10.1038/s44325-025-00086-2

**Published:** 2025-10-06

**Authors:** Be’eri Niego, Christoph E. Hagemeyer, Bianca Jupp, Asif Noor, Paul S. Donnelly, Rong Xu, Thirimadura V. H. Mendis, Yi Ching Chen, Irena Carmichael, Julie R. McMullen, Karen Alt

**Affiliations:** 1 NanoBiotechnology Laboratory, Australian Centre for Blood Diseases, School of Translational Medicine, Monash University, Melbourne, VIC Australia; 2 Monash Biomedical Imaging, Monash University, Melbourne, VIC Australia; 3 Department of Neuroscience, School of Translational Medicine, Monash University, Melbourne, VIC Australia; 4 School of Chemistry and Bio21 Molecular Science and Biotechnology Institute, University of Melbourne, Melbourne, VIC Australia; 5 Heart Research Institute, The University of Sydney, Sydney, NSW Australia; 6 Monash Micro Imaging, Alfred Research Alliance, Monash University, Melbourne, VIC Australia; 7 Baker Heart and Diabetes Institute, Melbourne, Victoria, Australia; Monash University, Melbourne, VIC Australia; 8 NanoTheranostics Laboratory, Australian Centre for Blood Diseases, School of Translational Medicine, Monash University, Melbourne, VIC Australia

**Keywords:** Imaging, Cardiology, Cardiovascular diseases

## Abstract

Atrial fibrillation (AF), often accompanied by atrial fibrosis, is challenging to diagnose sub-clinically and reverse once established. Molecular imaging targeting excess atrial collagen may enable earlier detection of atrial fibrosis and its associated arrhythmias. We used the collagen I-binding peptide EP-3533 and our novel ‘T-peptide’ targeting matrix metalloproteinases-2-digested collagen IV to image interstitial atrial fibrosis, AF and heart failure (HF) that develop in the double transgenic mouse model dnPI3K-Mst1 (termed ‘AF + HF’). Ex vivo and in vivo imaging were performed using near-infrared scans and positron emission tomography (PET) with probes conjugated to Cyanine5.5 and copper-64, respectively. Both tracers significantly accumulated in fibrotic atria compared to non-transgenic controls, with specific T-peptide uptake relative to a mutated ‘S-peptide’. Pharmacokinetic profiling demonstrated good tracer plasma stability and fast renal clearance. These results highlight the potential of collagen-targeted peptide tracers, particularly the disease stage-sensitive T-peptide, to improve diagnosis and monitoring of atrial fibrosis and AF.

## Introduction

Cardiac arrhythmias have reached epidemic levels around the globe^1^. In particular, hospitalisation due to atrial fibrillation (AF), the most common cardiac arrhythmia, has almost doubled over the past 30 years and its burden has surpassed other cardiovascular conditions, such as myocardial infarction (MI) and heart failure (HF)^2^. Atria-originated thrombi (resulting from turbulence or stasis in atrial blood flow) can lodge into the brain and cause dangerous strokes, nearly twice as fatal and twice as disabling as non-AF-related strokes^3^.

One of the key pathological processes accompanying AF is atrial fibrosis, which promotes the development of AF by altering the electrical conductivity in the heart and via creation of pathological atrial tissue more likely to develop and sustain the arrhythmia^4–7^. Of note, AF is also associated with the presence of diffuse left ventricular (LV) fibrosis^8^, which is indicative of adverse cardiac remodeling and poor clinical outcomes in HF and several other cardiac disease states^9^. Diagnosis of atrial fibrosis could therefore be of significant clinical importance.

Several collagen types were shown to be upregulated during human heart disease, including the extracellular matrix (ECM) collagens types I and III, and the basement membrane collagen type IV^10,11^. Additionally, the enhanced activity of collagen-modifying enzymes, such as matrix metalloproteinases (MMPs), has been demonstrated in cardiomyopathies^12,13^. Cardiac collagens and their digested forms are thus promising targets for the homing of imaging agents and therapeutics into affected heart tissue.

Notably, current ‘gold standard’ cardiac fibrosis imaging techniques, like late gadolinium enhancement (LGE) of the atrial/ventricular wall and T1 mapping on cardiac MRI (cMRI), are indirect and rely on the abnormal interstitial expansion, but not on the fibrotic matrix itself^14–17^. Moreover, they are limited by spatial resolution and image quality, especially in AF due to the thin atrial wall^14,18^, and suffer from uncertain reproducibility and a high degree of operator dependence^15,19^. Targeted imaging of atrial fibrosis using specific collagen tracers may overcome these limitations by enrichment of fibrotic atrial tissue with tracer-bound contrast agent, allowing a better signal to noise ratio and more accurate demonstration of the atrial condition^14^. Molecular approaches could thus deliver a much-needed improvement in early diagnosis and monitoring of atrial disease.

Here, we report an innovative use of collagen peptide tracers for detection of atrial fibrosis and diffuse, interstitial ventricular fibrosis in a well-characterized mouse model of atrial myopathy and AF, which develops HF over time^20,21^. The dnPI3K^Tg+/−^,DCM^Tg+/−^ double transgenic mouse (referred to hereafter as the ‘AF + HF’ mouse, in-line with our prior publications^21^) has a dual introduction of cardiac-specific, dominant negative phosphoinositide 3-kinase (dnPI3K) and Mammalian sterile 20-like kinase 1 (Mst1). These mice develop interstitial cardiac fibrosis over ~4–6 months, which is accompanied not only by dilated cardiomyopathy (DCM) and depressed cardiac function, but also by AF, atrial enlargement, atrial thrombosis and atrial fibrosis^20,21^. The ‘AF + HF’ mice are therefore ideal for examination whether collagen tracers are capable of molecular imaging of atrial myopathy and its associated cardiac disease.

Two collagen-targeting peptide tracers were employed in this study, the ‘T-peptide’ (TLTYTWS) that selectively binds matrix metalloproteinase-2 (MMP-2)-degraded collagen IV^22^, and the cyclic-peptide EP-3533^23,24^, which targets collagen I. Both tracers were conjugated to the near-infrared (NIR) fluorophore sulfo-Cyanine5.5 (Cy5.5) for ex vivo imaging, and the T-peptide also to the copper-64 (^64^Cu) ligand MeCOSar (5-(8-methyl-3,6,10,13,16,19-hexaaza-bicyclo[6.6.6]icosan-1-ylamino)-5-oxopentanoic acid) for in vivo imaging using positron-emission tomography (PET)^25^.

We recently used the T-peptide both ex vivo^26^ and in vivo^27^ to image the widespread interstitial and perivascular fibrosis that develops in the hearts of beta-2 adrenergic receptor (β2-AR) overexpressing mice (model of cardiomyopathy and HF)^28,29^. EP-3533 was previously used for MRI enhancement of type I collagen in murine models of myocardial infarction (MI)^23,24^, liver^30^ and pulmonary fibrosis^31^. The use of collagen peptide probes for the challenging detection and quantitation of diffuse atrial fibrosis, however, has never been described. Our results demonstrate the capacity of molecular imaging approaches based on collagen-targeted probes, in particular the T-peptide, to detect atrial fibrosis in vivo in male and female ‘AF + HF’ mice, highlighting their potential of becoming a new, valuable imaging tool for atrial disease.

## Results

### ‘AH + HF’ mice develop a widespread interstitial cardiac fibrosis, including upregulation of collagen types I and IV, and activation of MMP-2

**Fig. 1:**
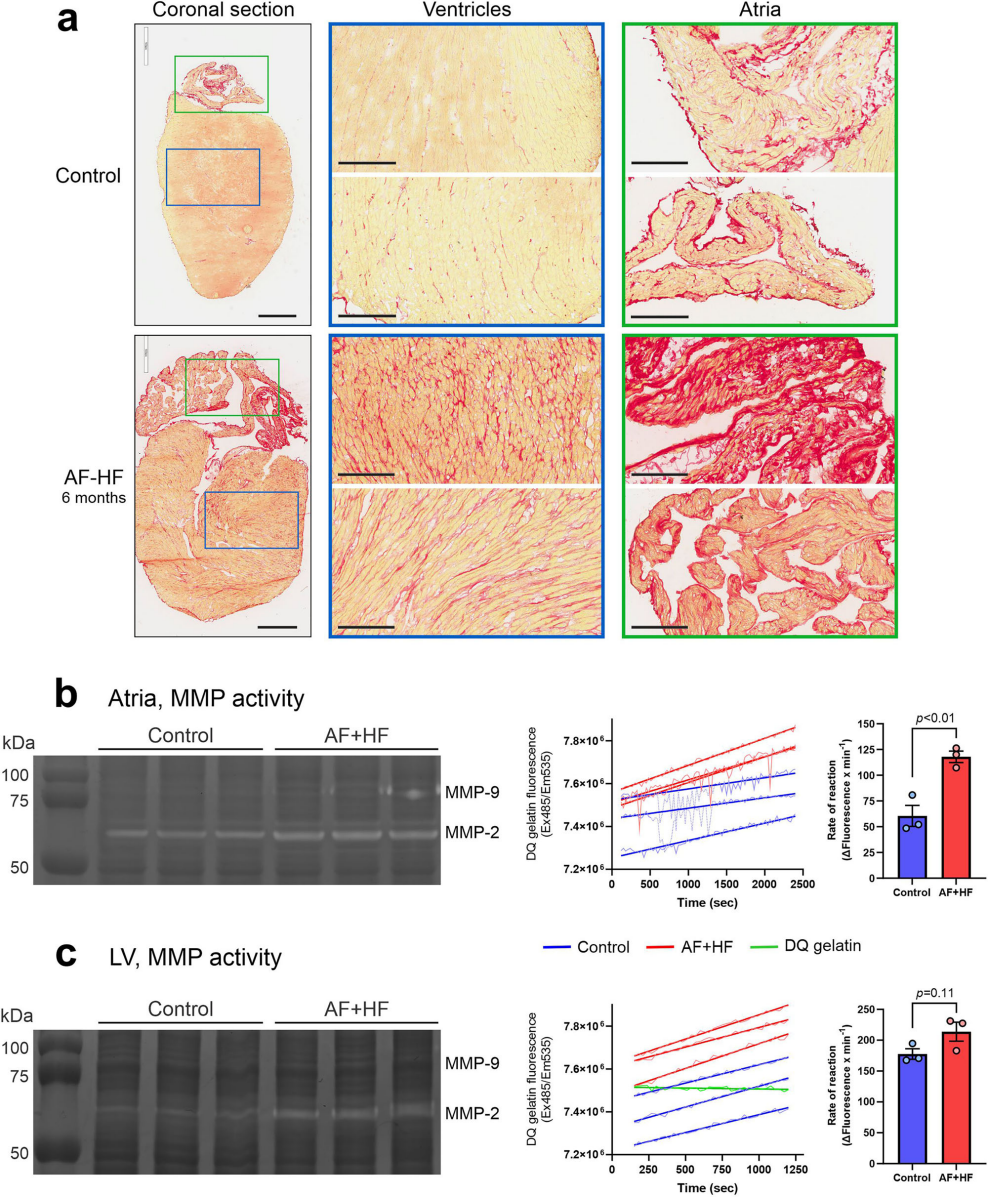
Interstitial fibrosis and active matrix metalloproteinases (MMPs) are enhanced in the ventricles and atria of the ‘AF + HF’ mouse model. **a** Representative images of Picrosirius red-stained coronal heart sections from ‘AF + HF’ double transgenic mouse (lower panels) and a healthy littermate control (upper panels) (6 months). The magnified blue-framed ventricular images (middle) and green-framed atrial images (right) correspond to the colored frames on the whole section (left). A wide spread interstitial fibrosis in both heart compartments – a hallmark of the ‘AF + HF’ mouse model^7,20^ - is evident. The significant expansion of atrial collagen, together with atrial fibrillation (AF), makes the ‘AF + HF’ mouse model suitable for molecular imaging studies of atrial disease. **b**, **c** Gelatin zymograms (left panels), as well as DQ™-gelatin-based MMP activity assays (curves in the middle panels and quantification of reaction rates in the right panels) on atrial (**b**) and left ventricle (LV) (**c**) heart lysates from ‘AF + HF’ and control mice (males; 28–29 weeks). An increase in the MMP-2 content and activity is observed in both heart chambers. These conditions could create an optimal target for the T-peptide probe that targets MMP-2-digeted collagen IV. *n* = 3. Mean±SEM. *p*-value as indicated by two-tailed Student’s *t* test. Scale bars in (**a**): left panels = 900 µm; middle and right panels = 200 µm.

The ‘AF + HF’ double transgenic mouse model was previously reported to develop a complex atrial disease and cardiomyopathies over 4–6 months (including AF and HF) that are accompanied by interstitial cardiac fibrosis in all 4 chambers of the heart^20,21,32^. Additionally, several collagen types and MMP-2 activation – all critical features for EP-3533 and T-peptide studies - were upregulated in these transgenic hearts^20,21,32^, making the ‘AF + HF’ strain a valuable tool for our imaging purposes. Indeed, Picrosirius Red (PSR) staining of coronal heart sections from transgenic mice and non-transgenic controls confirmed the presence of a wide-spread interstitial fibrosis in both the transgenic ventricles and atria at 6 months of age (Fig. 1a), with particularly thick deposits of collagen in atrial tissue (Fig. 1a, right panels). Assessment of the cardio-inflammatory state of these hearts by a gelatine zymogram showed a qualitative increase of MMP-2 in the upper and lower heart chambers of the transgenic mice relative to non-transgenic controls (Fig. 1b, c, respectively, left panels). Quantification of cardiac MMP activity in the atria and ventricles by a DQ™-gelatin based cleavage assay over time (Fig. 1b, c, middle panels) identified a significant 94.8% increase in reaction rate in transgenic atrial lysate (AF + HF 117.9 ± 5.6 *vs*. control 60.5 ± 10.2 (Mean ± SEM); Fig. 1b; right panel; *p* < 0.01) and a similar 20.3% upward trend in the transgenic ventricles (AF + HF 213.8 ± 15.5 vs. control 177.6 ± 8.5; Fig. 1c; right panel; *p* = 0.11). These results indicate a higher state of cardiac inflammation in the transgenic animals.

**Fig. 2:**
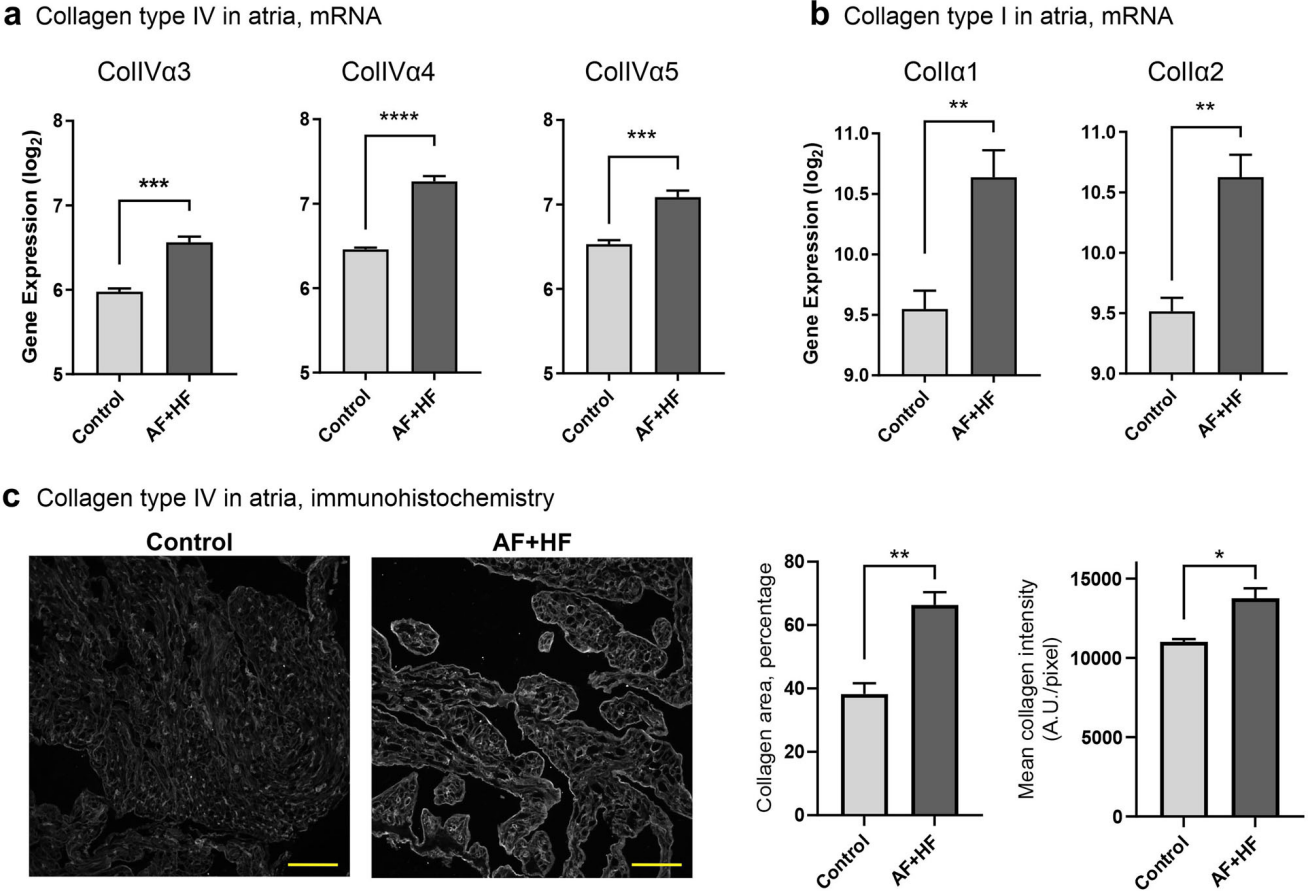
Collagen type I and type IV are upregulated in the hearts of ‘AF + HF’ mice. Gene expression microarray analysis demonstrating upregulation of the collagen IV alpha chains α3, α4 and α5 (**a**) and the collagen I α1 and α2 chains (**b**) in fibrotic atria of ‘AF + HF’ double transgenic mice relative to non-fibrotic control mice at 4.5-5 months of age. **c** Representative images of collagen IV-stained atrial tissue sections from control and ‘AF + HF’ mice (28–30 weeks; left panels), and quantification of the collagen-occupied area (middle), and mean collagen signal intensity (right) in these sections. This analysis confirms an increase in collagen IV in ‘AF + HF’ mouse atria. The ‘AF + HF’ mouse therefore contains the relevant types of cardiac collagens to suit molecular imaging with EP-3533 and the T-peptide tracer. *n* = 4 in (**a**, **b**), *n* = 3–4 in (**c**). Mean ± SEM. *p < 0.05, **p < 0.01, ***p < 0.001, ****p < 0.0001 by two-tailed Student’s *t* test.

To verify the presence of the relevant collagen types in the ‘AF + HF’ mouse model, we evaluated collagen mRNA expression in our microarray data from the atria of AF + HF mice^20^. A clear upregulation of alpha chains 3, 4 and 5 of collagen IV was observed in ‘AF + HF’ mice relative to controls (Fig. 2a; *p* < 0.001), as well as an increased expression of the collagen I alpha chains 1 and 2 (Fig. 2b; *p* < 0.01). We then confirmed the mRNA data for collagen IV by immunohistochemistry (Fig. 2c) and found an increase in both the collagen IV-covered atrial area (Fig. 2c; middle panel, *p* < 0.01) and mean pixel intensity (Fig. 2c; right panel, *p* < 0.05). These results indicate that collagens IV and I are among the collagen types that accumulate in the ‘AF + HF’ mouse heart, particularly the atria, providing an abundance of suitable substrate for our tracers to bind to.

### Pharmacokinetic and plasma stability profiles of the collagen-targeted peptide tracers

While the collagen tracers were previously found to be fast-clearing in β2-AR transgenic mice^26^, it was important to determine their clearance rate also in the ‘AF + HF’ mouse strain. Blood clearance assessment in a mixed cohort of male and female, transgenic and non-transgenic mice demonstrated a Cy5.5-EP-3533 circulating half-life of 11.1 ± 2.3 min (Supplementary Fig. 1a, b). Only 4.7% of the initial tracer intensity was detected 4 h post-administration (Supplementary Fig. 1c; *p* < 0.0001), suggesting that the tracer blood pool has largely been cleared by this time (when the ex vivo biodistribution studies with fluorescent peptides were performed; see below). Similar pharmacokinetic assessment for Cy5.5-T-peptide yielded blood half-life of 9.23 ± 0.9 min (Supplementary Fig. 1d). Finally, a plasma stability study has shown that 60% of the fluorescent tracer remained intact after 60 min of incubation in mouse plasma, but only 22% by 4 h post-incubation (Supplementary Fig. 1e). These data indicated that the fluorescent peptide probes are suitable for in vivo work as they rapidly clear (reducing their toxicity), but possess sufficient plasma stability to bind their substrates before degradation.

### The collagen I-targeted probe EP-3533 enhances the fibrotic atria in ‘AF + HF’ mice

**Fig. 3:**
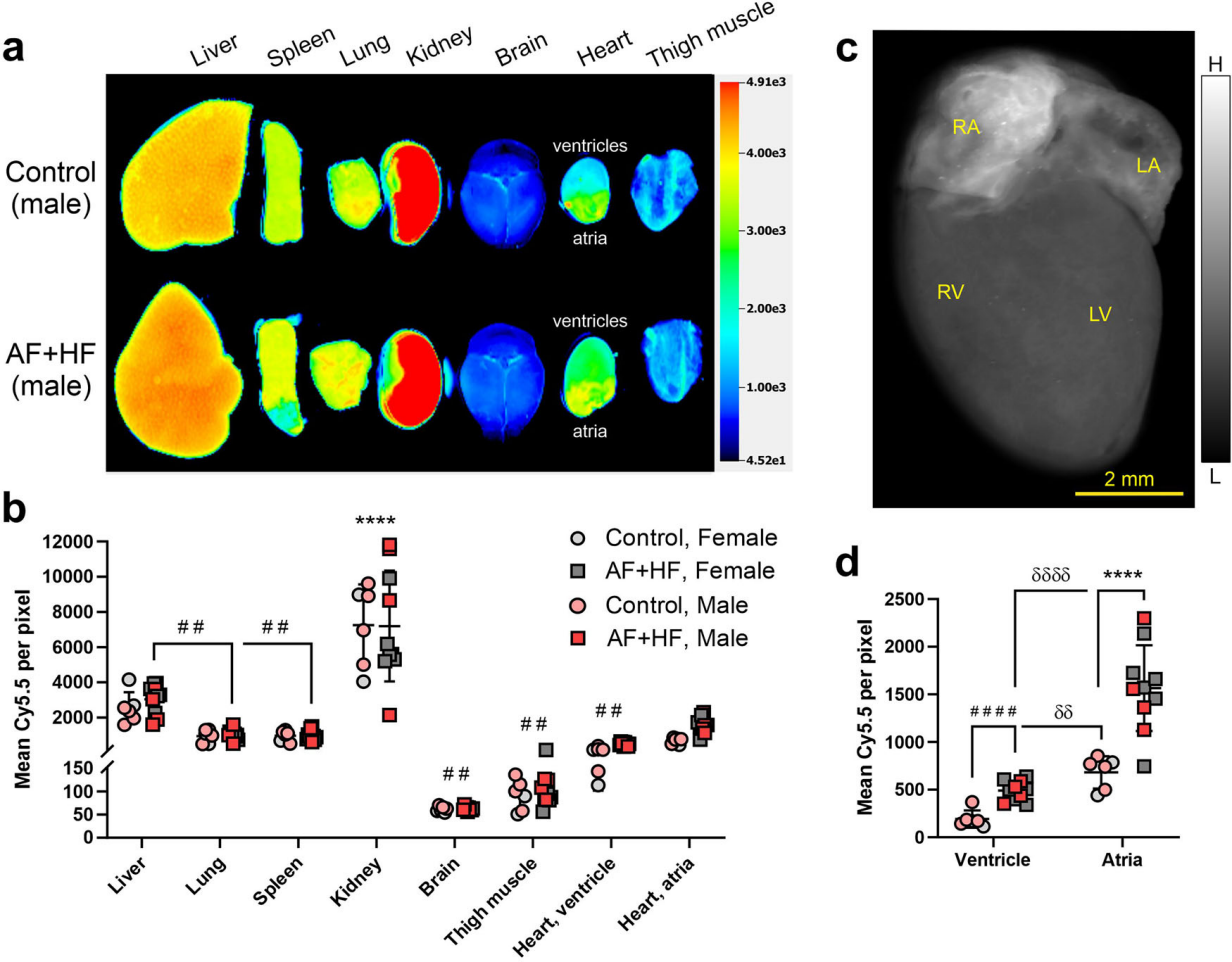
Ex vivo imaging of atrial fibrosis in ‘AF + HF’ mice with the collagen I-targeted cyclic peptide probe EP-3533. **a** A representative ex vivo near-infrared scan and **b** quantification of mean fluorescence intensity demonstrating the biodistribution of Cy5.5-conjugated EP-3533 (0.5 mg/kg) in perfused organs of double transgenic ‘AF + HF’ mice (29–31 weeks) and littermate controls at 4 h post-intravenous administration. Tracer uptake is similar between transgenic and control animals in all organs, except the heart (both in ventricles and atria). The prominent renal uptake indicates a renal clearance pathway. **c** A high-resolution, near-infrared ex vivo scan of an ‘AF + HF’ mouse heart (arbitrary color scale) and **d** a sub-analysis of the cardiac data from **b** showing robust tracer uptake by fibrotic atria, especially in the upper right chamber (RA). No significant differences between sexes are observed. Mean ± SD. *n* = 6 for control and *n* = 10 for ‘AF + HF’ (male mice in pink/red, female mice in gray). In (**b**) ****p < 0.0001 comparing the kidney to all other organs within each genotype; ^##^*p* < 0.01 comparing to the liver within each genotype, or as indicated, by two-way ANOVA with Sidak’s post-hoc. In (**d**) ****p < 0.0001 by two-way ANOVA with Sidak’s post-hoc; ^δδ^*p* < 0.01, ^δδδδ^*p* < 0.0001 by repeated-measure 2-way ANOVA with Sidak’s post-hoc; ^####^*p* < 0.001 by two-tailed Student’s *t* test, as indicated. Scale bar as specified. LA left atrium, LV left ventricle, RA right atrium, RV right ventricle.

Having confirmed that the ‘AF + HF’ mouse model and the targeted collagen tracers are well-suited for purpose, we next sought to validate the collagen-targeted imaging methodology by using the well-characterized, collagen I-targeted cyclic peptide EP-3533^23,24,30,31^. We therefore intravenously injected EP-3533, Cy5.5-labeled (see structure in Ezeani et al.; 0.5 mg/kg)^26^, and compared tracer accumulation in excised organs at 4 h post-administration (Fig. 3a). A two-way ANOVA analysis of the biodistribution (Fig. 3b) demonstrated that tissue accumulations of the cyclic-peptide were significantly dependent on the organ (F(7) = 73.53, *p* < 0.0001), but not the genotype, with no significant interaction between these factors (Fig. 3b). In particular, EP-3533 was highly concentrated in the kidney in both transgenic and non-transgenic mice (*p* < 0.0001 relative to all other organs by post-hoc analysis; Fig. 3b) and showed an increased uptake also in the liver (*p* < 0.01 relative to several organs; Fig. 3b). These results primarily indicate renal and some hepatic clearance pathways. Notably, high EP-3533 fluorescence levels were observed in the transgenic atria, which were comparable to the levels seen in the liver (Fig. 3b). No differences in tracer uptake were seen between male and female mice in both genotypes (Fig. 3a, b).

A sub-analysis focusing on EP-3533 uptake in the heart alone while comparing ventricles and atria (Fig. 3c) found peptide accumulation patterns that were significantly influenced by the genotype (F(1) = 34.77, *p* < 0.0001), the heart compartment (F(1) = 61.07, *p* < 0.0001) and their interaction (F(1) = 8.534, *p* = 0.0068) (Fig. 3d). Multiple comparison post-hoc tests showed a significantly elevated peptide levels in the transgenic atria relative to the non-transgenic controls (2.3-fold; *p* < 0.0001; Fig. 3d) and an enhanced uptake also in the transgenic ventricles (2.55-fold; *p* = 0.087 by two-way ANOVA with Sidak’s post-hoc; *p* < 0.0001 by *t*-test; Fig. 3d). As could also be observed in the image (Fig. 3c), a paired analysis confirmed that atrial uptake was significantly higher than ventricular not only in ‘AF + HF’ (3.2-fold; *p* < 0.0001), but also the in non-transgenic mice (3.55-fold; *p* < 0.01) (Fig. 3d). Within each group (tested independently), there was no variation in cardiac peptide intensity signals between male and female mice irrespective of the genotype. These analyses indicate that EP-3533 uptake is overall increased in the fibrotic ‘AF + HF’ heart, but that the fibrotic atrial compartment is particularly enhanced by this collagen-I targeted imaging approach. Nevertheless, broader differences between atrial and ventricular tissue might account for some of this enhanced atrial binding.

### The T-peptide strongly depicts fibrotic atria and ventricles in ‘AF + HF’ mice in a specific manner

**Fig. 4:**
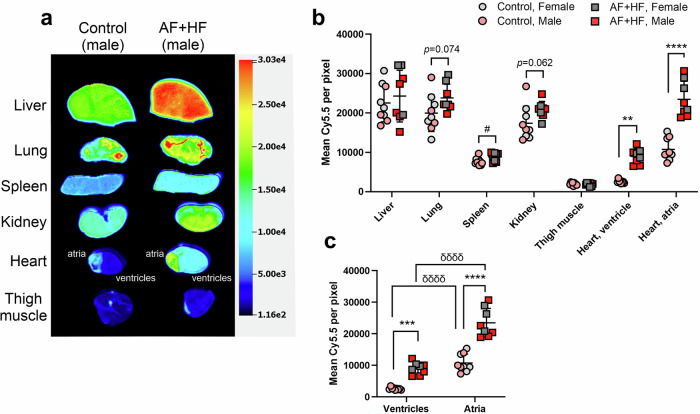
Ex vivo imaging with the T-peptide probe targeting MMP-2-digested collagen IV demonstrates atrial fibrosis in ‘AF + HF’ mice. **a** A representative ex vivo near-infrared scan and **b** quantification of the mean fluorescence intensities demonstrating the biodistribution of Cy5.5-conjugated T-peptide (0.5 mg/kg; 4 h post-intravenous administration) in perfused organs of double transgenic ‘AF + HF’ mice (28–30 weeks) and littermate controls. Tracer uptake is high in the liver, lung and kidney. The fibrotic atria are particularly enhanced to the levels seen in the liver. Significant uptake is observed in both the fibrotic heart compartments (ventricles and atria) relative to control. **c** A sub-analysis of the cardiac data alone showing robust tracer uptake by the fibrotic atria of ‘AF + HF’ mice relative to both the healthy atria and diseased ventricles. Mean ± SD. *n* = 9 for control and *n* = 8 for ‘AF + HF’ (male mice in pink/red, female mice in gray). In (**a**) and (**b**) **p < 0.01, ***p < 0.001, ****p < 0.0001 by two-way ANOVA with Sidak’s post-hoc; ^#^*p* < 0.05 and exact *p*-values by two-tailed Student’s *t* tests. In (**c**) ^δδδδ^*p* < 0.0001 by repeated-measure 2-way ANOVA with Sidak’s post-hoc.

We subsequently performed similar experiments with our Cy5.5-conjugated T-peptide tracer (see structure in Ezeani et al.^26^) that targets MMP-2-degraded collagen IV^22^ (0.5 mg/kg for 4 h; Fig. 4a). The ex vivo biodistribution two-way analysis showed significant variations originating from both the genotype (F(1) = 46.04, *p* < 0.0001), the organ (F(6) = 108.5, *p* < 0.0001) and their interaction (F(6) = 7.027, *p* < 0.0001) (Fig. 4b). Post-hoc analysis showed prominent T-Peptide accumulations in both transgenic and non-transgenic livers, lungs and kidneys, which were independent of the mouse sex (Fig. 4b). There were significantly higher tracer levels in transgenic spleens relative to healthy controls (*p* < 0.05), with similar trends developing also in lungs and kidneys (*p* = 0.074 and *p* = 0.062, respectively; Fig. 4b). Importantly, T-peptide concentrations were significantly elevated in the ‘AF + HF’ transgenic ventricles and most notably the atria (*p* < 0.01 and *p* < 0.0001, respectively; Fig. 4b). These results indicate an excellent tracer targeting to the fibrotic heart, particularly to collagen-rich atria, with primarily hepatic and renal clearance in both genotypes. Emerging trends for enhanced tracer uptake also in other transgenic organs could point to a broader fibrotic pathology in ‘AF + HF’ mice due to the failing heart.

To gain more detailed insights of the cardiac T-peptide uptake, we analyzed separately the fluorescent signals in both the lower and upper heart chambers of transgenic and non-transgenic mice (Fig. 4c). A significant enhancement of signal in the fibrotic atria and ventricles was observed in transgenic mice relative to the healthy controls (2.19-fold (*p* < 0.0001) and 3.49-fold (*p* < 0.001), respectively; Fig. 4c). As previously observed with EP-3533 (Fig. 3), a matched analysis further showed that the T-peptide retention in atrial tissue was significantly higher than the ventricles irrespective of the genotype, with 2.61- and 4.17-fold increase (atria/ventricles) in ‘AF + HF’ mice and their non-transgenic littermate controls, respectively (*p* < 0.0001; Fig. 4c). Taken together, the T-peptide was capable of strongly demonstrating the fibrotic ventricles and atria in the ‘AF + HF’ mouse ex vivo, with especially robust uptake in the fibrotic atrial compartment. Atrial peptide accumulation, however, seemed to have a significant basal component that was independent of the transgenic phenotype.

**Fig. 5:**
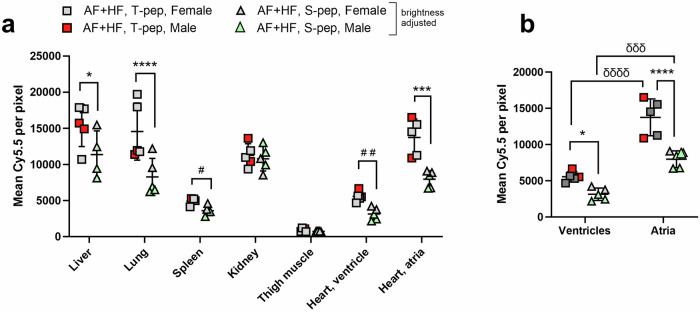
T-peptide uptake in the fibrotic atria of ‘AF + HF’ mice is specific. Comparison of the biodistribution in all major organs (**a**) and a sub-analysis of the cardiac accumulation (**b**) of Cy5.5-conjugated T-peptide (0.5 mg/kg; 0.258 µM/kg) *vs*. Cy5.5-labeled, mutated S-peptide (0.466 mg/kg; 0.258 µM/kg) in ‘AF + HF’ double transgenic mice (28–30 weeks). Perfused organs were scanned ex vivo on a near-infrared scanner 4 h post-intravenous tracer administration. The S-peptide signal was adjusted by the S-peptide/T-peptide brightness per mole slope ratio obtained from standard curves (Supplementary Figure 2). The T-peptide cardiac uptake is higher than the S-peptide in both ventricles and atria of ‘AF + HF’ animals, indicating its specificity. Mean ± SD. *n* = 5 per group (male mice in red squares and green triangles, female mice in gray). In (**a**) *p < 0.05, ***p < 0.001, ****p < 0.0001 by 2-way ANOVA with Sidak’s post-hoc; ^#^*p* < 0.05, ^##^*p* < 0.01 by multiple unpaired t-test adjusted for multiple comparisons by Holm–Sidak post-hoc. In (**b**) *p < 0.05, ****p < 0.0001 by two-way ANOVA with Sidak’s post-hoc; ^δδδ^*p* < 0.001, ^δδδδ^*p* < 0.0001 by repeated-measure 2-way ANOVA with Sidak’s post-hoc, as stipulated.

To further assess the specificity of the T-peptide binding to its substrate, we compared administration of the T-peptide (TLTYTWS; 0.5 mg/kg) and a mutated S-peptide (GLGYGWS; 0.466 mg/kg) in ‘AF + HF’ transgenic mice on an equimolar basis (0.258 µmol/kg; Fig. 5). Notably, we previously reported no differences in T- and S-peptide uptake in the hearts of healthy mice (β2-AR littermate controls)^26^. After correcting the S-peptide for brightness differences (as it was 2.5-fold brighter than the T-peptide mole-per-mole; Supplementary Figure 2), significant biodistribution variations arose from the organ (F(6) = 56.46, *p* < 0.0001), the peptide type (F(1) = 37.63, *p* < 0.0001) and their interaction (F(6) = 3.888, *P* = 0.0026) (Fig. 5a). A post-hoc test identified that S-peptide uptake was significantly lower than the T-peptide not only in the fibrotic atria (*p* < 0.001) and ventricles (*p* < 0.01 by *t*-test), but also in the liver (*p* < 0.05), lung (*p* < 0.0001) and spleen (*p* < 0.05 by *t*-test) (Fig. 5a). The levels of both peptides in the kidney remained comparable (Fig. 5a). Interestingly, there was a trend for reduced S-peptide binding in organs of male mice relative to females, which was not observed with the T-peptide (Fig. 5a). These data indicate that the T-peptide binding in the heart is specific, but that fibrotic changes may occur also in other ‘AF + HF’ mouse organs, particularly in males. A sub-analysis of the heart compartments alone confirmed a 1.72- and 1.78-fold higher T-peptide binding in atria (*p* < 0.0001) and ventricles (*p* < 0.05), respectively, relative to the S-peptide (Fig. 5b). However, S-peptide uptake in the atria was also 2.54-fold higher than the ventricles (*p* < 0.001, Fig. 5b), similar to the 2.47-fold seen with the T-peptide (*p* < 0.0001, Fig. 5b), suggesting once again an intrinsic tendency of the atria to absorb higher tracer levels.

### The T-peptide binds collagen-IV containing structures in the pectinate muscles of remodeled ‘AF + HF’ atria, which are also subjected to an increased MMP digestion

**Fig. 6:**
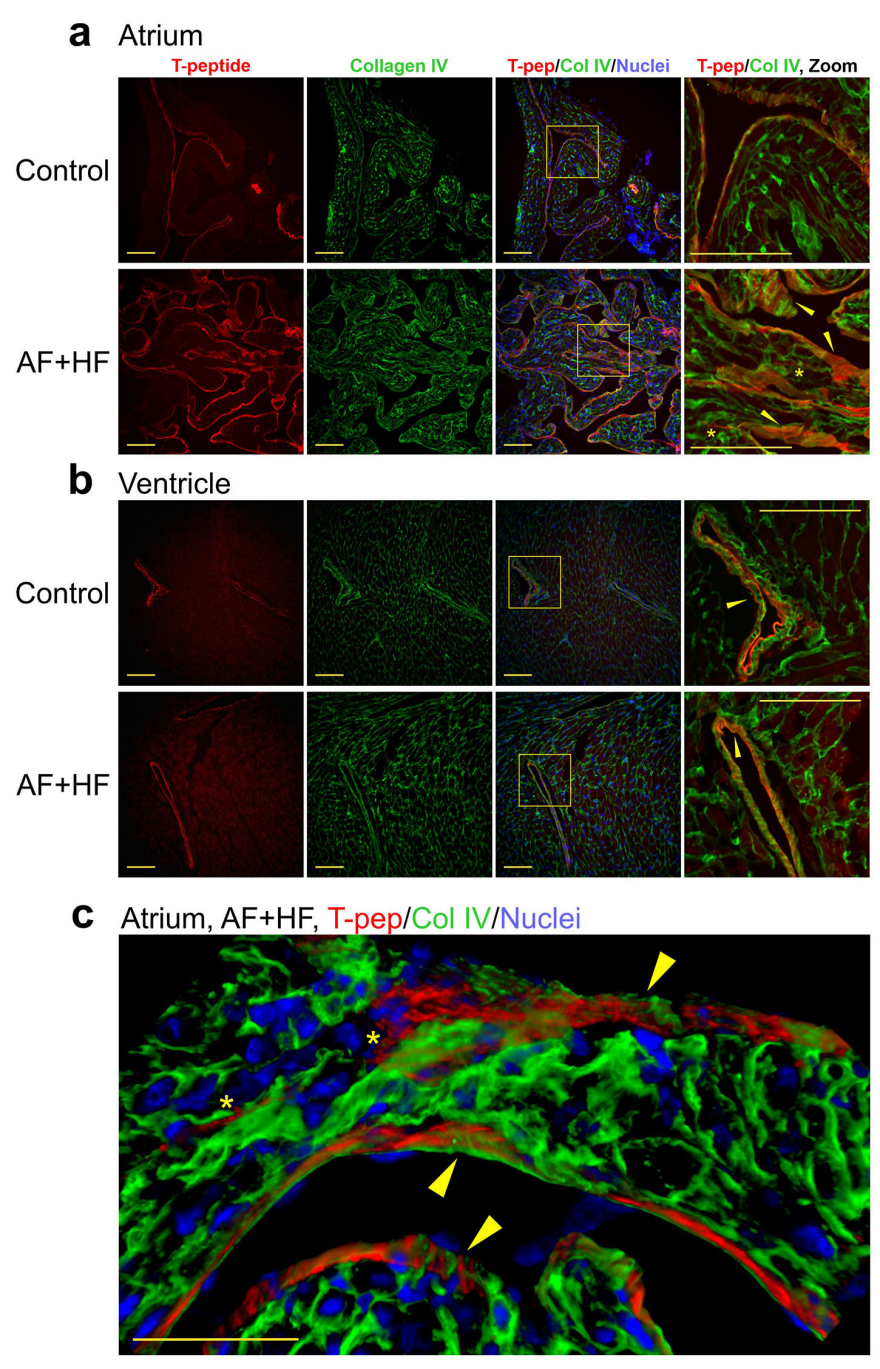
The T-peptide enhances collagen IV-containing structures in the atria of ‘AF + HF’ mice with distinct separation from intact collagen. Co-immunostaining for Cy5.5-labeled T-peptide (red) and intact collagen IV (green) in atrial (**a**) and ventricular (**b**) coronal sections of non-transgenic (control) and ‘AF + HF’ transgenic mouse hearts. The tracer labeling was performed in vivo by intravenous injection of fluorescent T-peptide (0.5 mg/kg; 4 h), followed by collagen IV immunohistochemistry ex vivo. Cell nuclei are depicted in blue (Hoechst). **a** In transgenic atria, the T-peptide strongly binds the thick fibrotic endocardium that envelopes the hypertrophic pectinate muscles (arrow heads), with some penetration into the atrial tissue (asterisks). The peptide staining pattern of fine filaments differs from the continuous pattern of intact collagen IV, and there is clear partitioning between the structures (right panels). **b** In ventricles, the T-peptide depicts the basement membrane of larger blood vessels, which are also positive for intact collagen IV (arrow heads); yet, while the two stains overlap, they do not co-localize. The right panels are magnifications of the framed areas in the adjacent panels. Scale bars = 100 µm. **c** A 3d reconstruction from a Z-stack of fibrotic ‘AF + HF’ atrial tissue (stained as described above). The tracer binds the thickened, fibrous atrial muscle endocardium (yellow arrow heads) and penetrates into the tissue (asterisks) in close contact with, but district separation from intact collagen IV. Scale bars = 50 µm.

Co-immunostaining of heart sections for intact collagen IV after in vivo labeling with Cy5.5-T-Peptide demonstrated bright T-peptide decoration of the thick, fibrous endocardial layer^33^ encapsulating the remodeled pectinate muscles within the right atria of ‘AF + HF’ mice, which also contained intact collagen IV (Fig. 6a, lower panels; arrowheads). Some T-peptide was also observed penetrating into the muscle in thin streaks (Fig. 6a, lower panels; asterisks), but the staining was generally localized to the external muscle surface, with limited enhancement of cardiomyocyte basement membranes. Staining was notably weaker in the non-transgenic atria and localized to the endocardial surface (Fig. 6a, upper panels). In the ventricles, the major structures positive for the T-peptide were larger vessels walls, which were also stained for intact collagen IV (Fig. 6b; arrowheads). The staining intensity of ventricular tissue did not dramatically differ between transgenic and non-transgenic hearts, in contrast to the atria, but the higher background and/or autofluorescence detected in ventricular tissue could have masked the effect (Fig. 6b). Interestingly, while T-peptide-labeled structures were also positive for collagen IV, there was a distinct partitioning between the two stains, with the T-peptide seemingly infiltrating between areas of intact collagen IV (Fig. 6c, arrowheads and asterisks; and Supplementary Movie 1). Furthermore, while the T-peptide staining presented as fine filaments in both atria and ventricles, the intact collagen IV staining pattern was thicker and more continuous (Fig. 6a, b, and Supplementary Movie 1). Interestingly, larger areas with more intense MMP activity were identified in the atria of transgenic animals by in situ gelatin zymography relative to control mice (Supplementary Fig. 3a; left and middle panels), with some MMP activity regions positioned in close proximity to T-peptide-stained endocardial structures (Supplementary Fig. 3a; right panels). In vitro studies then showed that exposure of human (Supplementary Fig. 3b) and mouse (Supplementary Fig. 3c) collagen IV to MMP digestion indeed renders it “stickier” for the T-peptide, but additional studies are required to solidify these observations. Taken together, these microscopic analyses suggest that the T-peptide concentrates in fibrotic, collagen IV-containing endocardial muscular structures within the remodeled transgenic atria, which are exposed to higher level of MMP digestion that may increase their capacity of T-peptide binding. Yet, the T-peptide attachment patterns do not fully overlap with intact collagen IV, suggesting that fundamental differences exist between these substrates.

### PET imaging with the T-peptide demonstrates fibrotic cardiac remodeling in ‘AF + HF’ mice in vivo

**Fig. 7:**
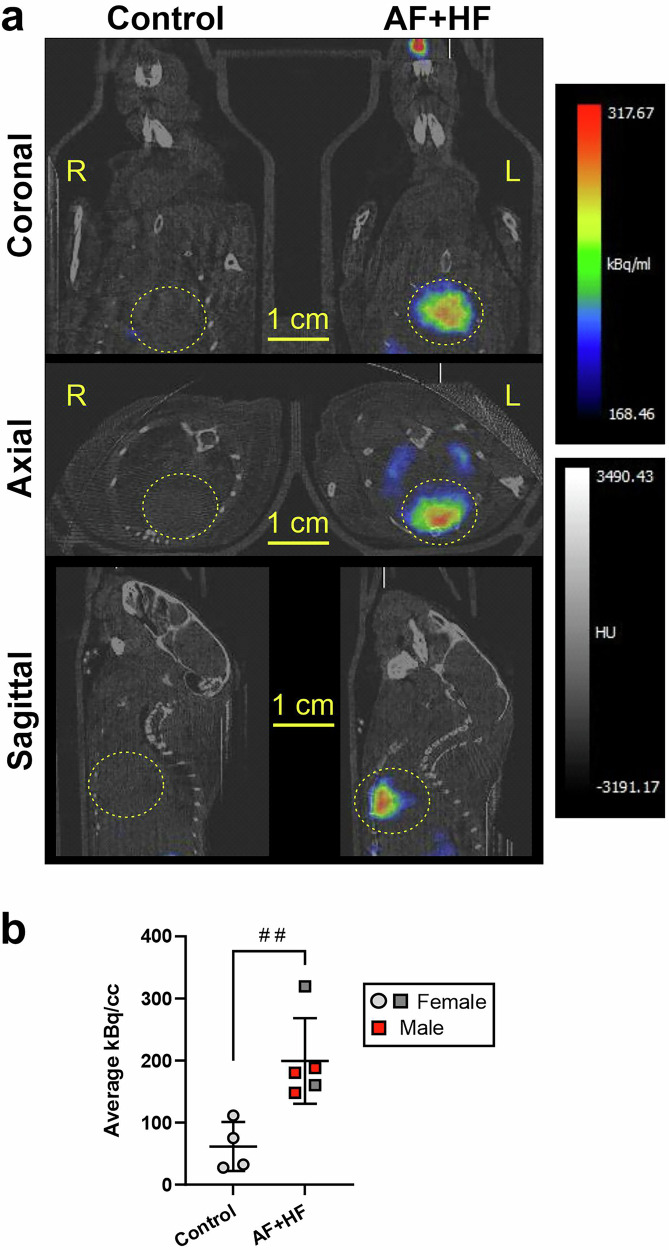
PET imaging with the T-peptide demonstrates cardiac fibrosis in ‘AF + HF’ mice in vivo. **a** Representative coronal, axial and sagittal PET/CT images of a control mouse (left) and an ‘AF + HF’ transgenic mouse (right) showing cumulative cardiac accumulation of ^64^Cu-T-peptide-MeCOSar^27^ (0.25 mg/kg; 25.86 ± 2.16 MBq) in the transgenic, but not control heart. The multi-bed PET acquisition was performed between 30 and 45 min post-tracer administration. **b** Quantification of the cumulative cardiac PET signal (in kBq/cc; acquisition parameters as in (**a**)) comparing ‘AF + HF’ mice to non-transgenic littermate controls (28–30 weeks). Strong enhancement of the transgenic (fibrotic) heart is observed relative to controls. Mean ± SD. *n* = 4 for control and *n* = 5 for ‘AF + HF’ (male mice in red, female mice in gray). ^##^*p* < 0.01 by t-test. Scale bars as indicated on the image. CT computed tomography, kBq/cc kilobecquerel per millilitre, MeCOSar 5-(8-methyl-3,6,10,13,16,19-hexaaza-bicyclo[6.6.6]icosan-1-ylamino)-5-oxopentanoic acid, PET positron emission tomography.

Lastly, we performed an in vivo PET study comparing ‘AF + HF’ transgenic mice and controls, using a ^64^Cu-labeled T-peptide-MeCOSar conjugate (see structure in Niego et al.^27^; 0.25 mg/kg; 25.86 ± 2.16 MBq). Analysis of the cumulative PET signal in the whole heart within 30-45 min post tracer-administration (Fig. 7a; multi-bed PET) demonstrated a significant 3.24-fold increase in T-peptide accumulation in vivo in transgenic hearts relative to controls (*p* < 0.01, Fig. 7b). Images from a single-bed CT also enabled a broad delineation of the upper and lower heart chambers (atria and ventricles, respectively), to estimate the radiation in each heart compartment from the overlayed PET/CT (Supplementary Fig. 4a; lower panels). This analysis identified elevated tracer levels in the upper left and right portions of the fibrotic ‘AF + HF’ heart (which we interpret as atria) relative to non-transgenic controls (4.2-fold in the right atrium (*p* < 0.01) and 3.6-fold in the left atrium (*p* < 0.05); Supplementary Fig. 4b). Tracer accumulation in the lower portion of the transgenic heart (i.e., the ventricles) was 3-fold greater than controls (*p* < 0.05) (Supplementary Fig. 4b). There was also a trend for higher tracer uptake in the right atrium relative to the ventricles in transgenic hearts (*p* = 0.074; Supplementary Fig. 4b), but a consistently low uptake in all chambers of the non-transgenic hearts (Supplementary Fig. 4b). While this interesting analysis is in-line with the fluorescent tracer data (Figs. 3 and 4), a motion-gated PET/CT scan is required for precise identification of the heart chambers to avoid signal bleeds and validate these chamber-to-chamber variations. Overall, these results prove the ability of T-peptide-based molecular PET imaging to detect cardiac fibrosis in a mouse model of atrial fibrillation and heart failure in vivo. Furthermore, a delayed and shorter acquisition period (30–45 min as compared to 4 h in the ex vivo studies) may be optimal to distinguish specific collagen binding from background noise, especially for imaging of atrial disease in vivo.

## Discussion

AF and other cardiac pathologies stemming from the atria (e.g., supraventricular tachycardia, atrial flutter) are prevalent conditions that are challenging to diagnose and may turn dangerous if not treated in time^7^. Here, using the ‘AF + HF’ double transgenic mouse model of cardiomyopathy and AF^20,21^, we showed that atrial fibrosis, which often accompanies arrhythmias, can be harnessed for molecular imaging of the diseased atria. In addition to validation of the imaging approach ex vivo with the well-characterized, collagen I-targeted peptide EP-3533, we successfully tested our new, smart T-peptide probe that targets MMP-2-digested collagen IV both ex vivo and in vivo using PET. The latter result is particularly exciting, since collagen turnover is thought to be high at the early stages of disease, when it is ‘active’ and developing^12,13^, and can still be reversed with appropriate interventions. Together with the high sensitivity of PET, molecular imaging with the T-peptide could represent a new and powerful approach for early detection and more effective treatment of AF. The potential clinical benefits are multiple, including precise characterization of abnormal tissues to optimize patient selection and guide treatment measures (e.g., ablation procedures in AF), detection of sub-clinical disease, improved prediction of complications, like stroke and cardiac arrest, and monitoring of intervention outcomes.

The ‘AF + HF’ mouse model chosen for this work has been previously shown to harbor most of the critical elements required for our imaging study, namely atrial and ventricular fibrosis, and upregulation of atrial collagen I and MMP-2 relative to non-transgenic littermate controls^20^. We confirmed the upregulation also of collagen IV in atrial tissue (Fig. 2), in line with clinical observations^10,11^, and verified the upregulation of MMP-2 in transgenic hearts *versus* control (with no noticeable difference in inducible MMP-9; Fig. 1), indicating that cardiac inflammation persists in the AF + HF model in late disease stages. These attributes make the ‘AF + HF’ model relevant and suitable for EP-3533 and T-peptide studies. Of note, our studies utilized older transgenic mice (5.2–6.5 months *versus* 4.5-5 months in the original report^20^) that displayed severe interstitial fibrosis throughout the myocardium and prominently in the atria (Fig. 1). Notably, there was detectible MMP activity also in control mice (Fig. 1), which could stem from normal cardiac collagen turnover processes and/or aging-dependent upregulation of MMP activity. While lower in intensity than transgenic mice, the MMP activity in control hearts most likely still contributed to a stronger T-peptide background binding under basal conditions. This implies that the conceptual advantage of the smart T-peptide probe was perhaps limited in our study. Indeed, both EP-3533 and the T-peptide enhanced the fibrotic *versus* healthy atrial image ex vivo to a similar degree (2.3- and 2.19-fold, respectively), suggesting that both approaches may be fit for purpose. Nevertheless, we believe that the ability of T-peptide-based imaging to resolve atrial differences may in fact be underestimated and the real dynamic range greater than observed. Future studies in younger animals at earlier stages of atrial disease and comparative quantification of the collagen types in the heart will help unravel the full potential in the use of each peptide probe for atrial disease delineation.

The potential advantage in using the T-peptide can in fact be inferred from the biodistribution data of organs other than the heart. Whereas for the collagen I tracer there was essentially no intra-organ variation between transgenic and control animals (Fig. 3), the tendency for enhanced T-peptide uptake was observed in several transgenic organs *versus* controls (Fig. 4). These findings are in line with the understanding that a failing heart also induces deterioration in other organs, implying that fibrotic changes could develop in additional tissues in ‘AF + HF’ mice. T-peptide imaging may therefore identify pathological changes that cannot be recognized by tracers for intact collagen (e.g., EP-3533), adding unique capabilities to its utilization. Yet, purposely-designed comparative studies will need to be crafted to test these possibilities.

To demonstrate T-peptide binding specificity, we used a control S-peptide and showed its reduced binding capacity compared to the T-peptide on a mole-per-mole basis (Fig. 5 and Ezeani et al.^26^). Furthermore, we previously blocked the radioactive T-peptide signal in the fibrotic hearts of β2-AR overexpressing mice with an excess of non-radioactive probe^27^. This evidence indeed indicates a specific component in T-peptide binding. However, some observations in our study will require further work; for example, peptide uptake was significantly higher in atria *versus* ventricles also in non-transgenic hearts (Fig. 4b) and in S-peptide-treated transgenic animals (Fig. 5b). Further, the T-peptide did not truly co-localize with intact collagen IV but displayed a different staining pattern, and also accumulated in areas with no obvious MMP activity on in situ zymography, as observed on immunohistochemical examination (Fig. 6 and Supplementary Fig. 3, respectively). It has long been known that the collagen content in the atria is higher than in ventricles under normal and pathological conditions (2-4-fold)^26,34,35^. Furthermore, the pectinate muscles’ structure, especially in hypertrophic atria and their fibrotic appendages^33^ (which is the hallmark of the ‘AF + HF’ mouse model^20^), create a ‘sponge-like’ structure that has much higher surface area to volume ratio than in healthy atria and ventricles. These properties not only provide abundant atrial substrate for the T-peptide to bind to, but also a greater surface for interaction with the muscle wall. Collectively, a combination of specific T-peptide binding, as well as physical peptide accumulation in the hypertrophic, collagenous and inflamed atrial structures, may contribute together to the higher peptide uptake levels seen in the ‘AF + HF’ transgenic atria. As for the lack of T-peptide and collagen IV co-localization, this could result from (1) competition of the T-peptide and the collagen IV primary antibody on similar collagen epitopes; (2) the increased T-peptide affinity towards MMP-2-digested collagen IV, which could differ in position to intact collagen, even within the same structure; and/or (3) technical artefacts due to the extensive washing required for ex vivo collagen IV immunohistochemistry on an in vivo T-peptide-labeled section. Another factor worth examining is the MMP content in the blood of ‘AF + HF’ mice, elevation of which could generally render all blood contacting surfaces ‘stickier’ for the T-peptide. While additional studies are required to ascertain all these fascinating mechanistic aspects of the T-peptide, the tracer was ultimately capable of distinguishing fibrotic from healthier atrial tissue, both ex vivo and importantly in vivo. For the latter, even though the upper portion of the heart appeared distinctly brighter on the PET/CT (Fig. 7 and Supplementary Fig. 4), indicative of a higher tracer uptake, a motion correction is required for precise identification of the atria and validation of the findings in vivo.

Encouragingly, the pharmacokinetic profiles of both peptide tracers and especially the plasma stability data of the T-peptide make feasible a future clinical translation of these molecular imaging approaches for AF. Both peptides were fast to clear (half-life of ~10 min; Supplementary Fig. 1a, c) and were previously shown to possess no inherent biological toxicity in the highly exposed liver and kidneys^26^. Importantly, while the tracers were almost completely cleared from the blood by 4 h post-administration, the T-peptide remained stable in plasma (60% intact after 60 min; Supplementary Fig. 1e). Such probe characteristics allow sufficient time for target interaction, as well as (limited) tissue penetration and retention. With particular relevance to our study design, these properties also ensured that the ex vivo images at 4 h almost entirely captured tissue-bound tracer (with no influence of the blood pool) and that the PET images at 30–45 min post-injection were largely generated from an intact T-peptide tracer. The overall profile emerging is of tracers that are quickly cleared, safe for use, but remain stable and potent in their target binding. While these elements are all crucial for potential translation of this new imaging methodology, the implementation of PET will require dosimetry studies to ensure that radiation levels remain within safe boundaries.

In summary, in this work we laid the foundations for deployment of molecular imaging with collagen-targeted tracers, especially PET with the innovative T-peptide, as a valuable tool aimed at sensitive diagnosis, monitoring and follow-up of AF via its associated atrial fibrosis. These capabilities expand the potential use of collagen-targeted medical imaging techniques not only to MI^23,24^, diffuse ventricular fibrosis and HF^26,27^, as we and other have shown, but also to prevalent and challenging-to-image cardiac conditions stemming from the atria. Future translation efforts of the T-peptide and other collagen probes in routine cardiac imaging will need to prioritise those indications for which capability gaps exist on cMRI and echocardiography. Compromised cardiac tissue likely develops subtle fibrotic abnormalities years before the manifestation of clinical symptoms, but early pathological changes are often beyond the detection limits of current imaging methodologies. Our molecular imaging approach, building on the great sensitivity of PET and direct collagen detection nature of the peptide tracers (as opposed, for example, to indirect LGE), should encourage clinical adoption of these new imaging methodologies. Newly added valuable capabilities, as early identification of fibrosis-associated ventricular disease and atrial arrhythmias by sensitive PET and molecular collagen probes, could prevent serious consequences of heart disease, such as stroke and cardiac arrest, enable patient stratification, guide personalized treatment and provide critical insights into the effectiveness of novel drugs. Careful patient selection and public cost considerations will have to be factored in when assessing the feasibility of implementing these probes into the clinic. Nevertheless, molecular imaging of cardiac conditions with collagen tracers like the T-peptide, especially of the atria, could make a substantial impact on cardiac patients and clinical practices.

## Methods

The data that support the findings of this study are either provided within the article, or available from the corresponding author upon a reasonable request.

### Animals

All animal studies were approved by Monash University’s Alfred Research Alliance (ARA) Animal Ethics Committee (AEC approval numbers E/1941/2019/M and E/1625/2016/M). Experimentation was carried out in accordance with the ‘Australian code for the care and use of animals for scientific purposes’, and reported in accordance with the ARRIVE guidelines. We used the well characterized heterozygote male and female dnPI3K^+/−^, DCM^+/−^ double transgenic (Tg) mice (dnPI3K^Tg+/−^, DCM^Tg+/−^, termed ‘AF + HF’ for simplicity, as previously described^21^) and non-transgenic littermate controls (dnPI3K^Tg−/−^, DCM^Tg−/−^; referred to as ‘Control’) at 23–31 weeks of age (5.1–6.9 months)^20,21^. Animals were bred by the Precinct Animal Centre of the ARA and housed in a temperature and humidity-controlled facility on a 12-h light/dark cycle, with ad libitum access to standard chow (Mice Maintenance Cube, Barastoc/Ridley, Australia) and water. Due to the low Mendelian frequency of double transgenic (dnPI3K-Mst1) male mice (~12.5%) and practical constraints related to breeding, we could obtain only a limited number of male and female mice, and combined them into larger groups. Nevertheless, earlier characterization studies of the ‘AF + HF’ model consistently demonstrated robust and reproducible atrial fibrosis phenotype in both sexes at the ages tested here^20,21,32^, making the mixed group approach suitable for this proof-of-principle imaging study.

### Peptides synthesis and conjugation

The ‘T-peptide’, a linear heptapeptide (sequence: TLTYTWS), was first identified as a tumor-homing peptide by phase display technology^22^. The T-peptide is unique in that it binds MMP-2-degraded collagen IV and has dual affinity towards both the mouse and human proteins^22^. To assess the T-peptide specificity, we used a control peptide termed ‘S-peptide’ (GLGYGWS)^26^. For validation of our methodology, we synthesized a version of the collagen I-targeted cyclic peptide EP-3533^23,24^, which was previously utilized by our group and others for cardiac fibrosis imaging studies^23,24,26^. All peptides were synthesised using a standard solid phase method and conjugated to the NIR fluorophore sulfo-Cy5.5 (excitation/emission 675/694 nm) to enable fluorescent imaging studies ex vivo, as we described (including a full molecular structure)^26^. For PET imaging, the T-peptide was conjugated to MeCOSar and labeled with ^64^Cu at a 1:2 ratio (peptide (µg):^64^Cu (MBq), as we recently detailed (including molecular structure)^27^. Using this method, we achieved radiochemical purity of 91% and decay-corrected crude radiochemical yield of 91%, resulting in crude product specific activity of 1.7 MBq/µg^27^.

### Ex vivo fluorescent imaging

Ex vivo fluorescent imaging was performed according to our published protocol^26^. In brief, fluorescently-tagged EP-3533 or the T-peptide were injected intravenously into mice at 0.5 mg/kg via the tail vein. The S-peptide was delivered at 0.466 mg/kg, being the identical mole equivalent to the T-peptide (0.258 µmol/kg). Injections were performed by a person blinded to the administered substance. Animals were humanely euthanised 4 h post-injection by an overdose of ketamine (300 mg/kg) and xylazine (30 mg/kg) and perfused via the heart with 20 ml of PBS. The liver, lung, spleen, kidney, brain, thigh muscle and the heart were next harvested and the atria separated from the ventricles. Organs were then scanned on the Odyssey CLx NIRF 2-D scanner (Li-Cor Biosciences, NE, USA) at 169 µm resolution using the ‘Manual L1’ or ‘L2’ intensity and 0.5 mm focus offset settings. A noninjected mouse served for background correction. The mean fluorescence intensity value per pixel (1 pixel = 169 × 169 µm = 0.0285 mm^2^) were then extracted from regions of interest using Image Studio 5.2 software (Li-Cor Biosciences). Notably, Since the Odyssey scanner generates 2D images, we used pixels (area) as a normalization method, instead of weight.

### In vivo PET imaging

Static PET scans were performed as we recently described^27^. Mice were injected via the tail vein with ^64^Cu-T-peptide-MeCOSar (10 µg of protein per animal; ~0.25 mg/kg; 25.86 ± 2.16 MBq (mean ± standard deviation (SD)). The animals were anaesthetized 30 min post-injection by isoflurane (4% induction, 1.5-2% maintenance, in 0.8 L/min oxygen) and placed in a nanoScan® PET/CT system (Mediso; Hungary). PET images were then acquired in ‘list’ mode for 15 min, followed by a 10 min CT (CT; acquisition parameters: X-ray voltage 35 kVp (680 µA); exposure time 300 ms; and a pitch of 1.0). 720 projections over 360° of rotation were acquired with data binning at 1:1 and then reconstructed using a filtered back projection algorithm with a Butterworth filter (voxel size 0.34 µm^3^). A single static PET image for the period of 30–45 min post-injection was then generated (voxel size 0.6 mm^3^; matrix 80 × 60 × 75) using the Tera-tomo 3d algorithm provided by the supplier, with correction for scatter, attenuation and dead-time. Images were finally calibrated in kilobecquerel per millilitre (kBq/mL).

### Histology and immunohistochemistry

4 h post-injection of Cy5.5-T-peptide, perfused hearts were excised, as stipulated above, and freshly frozen in Optimal Cutting Temperature (OCT) compound. Hearts were then sectioned at 6-µm thickness, in series. One series of sections was stained for picrosirius red, then imaged by the Aperio AT Turbo digital whole slide scanning system (Leica Microsystems, Australia) at x40 magnification and visualized with the Aperio ImageScope software (Leica Microsystems). For collagen IV immunohistochemistry^26^, sections (already labeled in vivo with Cy5.5-T-peptide) were first washed to remove the OCT, then incubated for 1 h at room temperature in blocking solution (10% goat serum and 0.1% TX-100 in Tris-buffered saline (TBS; 50 mM Tris, 150 mM NaCl, pH 7.6). An anti-collagen IV primary antibody (ab6586; 10 µg/ml; Abcam, Australia) in blocking solution (4% goat serum and 0.1% TX-100 in TBS) was applied overnight at 4 °C. Following 3 ×5 min wash with TBS, Alexa Fluor™ 488 goat anti-rabbit IgG secondary antibody (2 µg/ml; Thermo Fisher Scientific, Australia) was applied in blocking solution for 3 h at room temperature. Nuclei were counter stained with Hoechst (5 µg/ml; Thermo Fisher Scientific) for 1 h, added 2 h post-application of the secondary antibody. The sections were finally washed 3 ×5 min with TBS and mounted using ProLong™ Diamond Antifade mountant (Thermo Fisher Scientific). For colocalization studies, images were acquired on a Leica THUNDER widefield, inverted fluorescence microscope equipped with a HC PL APO CS2 20×/0.75 IMM UV multi-immersion lens and a Leica sCMOS K5 camera (Leica, Wetzlar, Germany). Image processing was performed using the Leica LAS-X software (version 3.0.16120) with Large Volume Computational clearing (LVCC) and deconvolution algorithm for thick samples (Leica). For collagen IV analysis, images were acquired on the THUNDER microscope using a HC PL FLUOTAR L 20×/0,40 PH1 CORR objective. Image processing was performed using the Nikon NIS-Elements software, Version 5.4 (Nikon, Tokyo, Japan). To quantify the total tissue area and collagen content, the 488 nm channel was segmented using two threshold levels: one to identify the tissue boundary and another to isolate the collagen signal. Binary images were generated from the threshold data, from which the total tissue area and total collagen area were calculated. Additionally, the mean intensity of the collagen signal was measured from the collagen-specific threshold data.

### Microarray analysis

mRNA expression data of collagen I and IV alpha chains were obtained by mining our previously published open-access microarray data set^20^. The complete raw and normalized array data are available through the Gene Expression Omnibus of the National Centre for Biotechnology Information (http://www.ncbi.nlm.nih.gov/geo/, accession number GSE12420).

### Blood clearance

‘AF + HF’ mice and non-transgenic controls (28–31 weeks) were intravenously injected with Cy5.5-labeled T-peptide or EP-3533 (0.5 mg/kg), as described above. Blood samples (2.5 µl) were collected from the opposite tail vein at 2–4, 12 and 240 min, and diluted in 47.5 µl of citrated PBS (0.32% w/v sodium citrate). 50 µl samples were transferred into 96-well plates (Optiplate, black-walled, transparent bottom; PerkinElmer, MA, USA) and scanned on the Odyssey CLx NIRF scanner using the ‘Manual L1’ intensity and 3 mm focus offset settings. Following subtraction of unlabeled blood background, the total fluorescence per well was plotted against time. The blood fluorescence levels at *t* = 0 and half-life were extrapolated from a one-phase decay non-linear regression, and the data was presented as percentage from the value of *t* = 0^26^.

### Mouse plasma stability

Cy5.5-T-Peptide (100 µg, 10 µL in PBS) was added to 200 µL of naïve C57BL/6 mouse plasma and the sample was incubated at 37 °C over 4 h. Aliquots (40 µL) were taken from the mixture at 5, 10, 30, 60 and 240 min, and then added to 40 µL of acetonitrile. The formed precipitates were separated by centrifugation and the supernatant was analyzed by HPLC. The percentage of decomposition at each timepoint was then calculated from the chromatograms (Supplementary Fig. 1). The HPLC conditions were: Phenomenex Luna® 5 µm C18(2) 100 Å, LC Column 150 ×4.6 mm at a flow rate of 1 mL/min (Method: 5–95% buffer B to A over 20 min, λabs = 280 nm).

### Gelatine zymography

MMP-2 and -9 levels were evaluated in ventricular and atrial lysates of perfused hearts from ‘AF + HF’ mice and controls (60 µg/lane), as we previously described^26^.

### In situ zymography on heart sections

In situ zymography was performed as described in Fukuta et al.^36^. In brief, 10 µm-thick fresh frozen heart sections were obtained from Cy5.5-T-peptide-treated ‘AF + HF’ and control mice, as detailed in “Histology and Immunohistochemistry” above. Sections were washed twice in TBS to remove the OCT compound. 0.1 mg/ml DQ™-gelatin (Thermo Fisher Scientific) in reaction buffer (TBS (see Histology above) with 5 mM CaCl_2_ and 0.2 mM Sodium Azide, pH 7.6) was then applied to each section (100 µl) for 24 h in a humidified chamber at 37 °C. Sections were then washed once in TBS and fixed with 4% (w/v) paraformaldehyde (PFA) supplemented with Hoechst (5 µg/ml) for 1 h at room temperature. Following another TBS wash, sections were finally coverslipped using ProLong™ Diamond Antifade mountant (Thermo Fisher Scientific). Immunofluorescence images were captured on the Leica THUNDER microscope and processed using the Leica LAS-X software (version 3.0.16120) with LVCC and deconvolution, as described above.

### DQ™-gelatin-based MMP activity assay in heart lysates

Net MMP activity in the heart chambers was evaluated in atrial and ventricular lysates from ‘AF + HF’ and control mice. Lysates were prepared by homogenization of fresh tissue in PBS with 0.1% (v/v) Triton-X100 at 250 wet mg/ml. Lysates were then clarified at 8000 × *g* for 10 min and the protein content quantified by the bicinchoninic acid (BCA) assay (Thermo Fisher Scientific). 60 µg of total protein per atrial sample and 100 µg of total protein per left ventricle sample were then mixed with DQ™-gelatin (0.1 mg/ml) in reaction buffer, as stipulated for the in situ zymography above, in a total volume of 200 µl per well. Fluorescein fluorescence (Excitation 483/Emission 530) was then measured on a CLARIOstar Plus multi-well plate-reader (BMG Labtech, Australia) in 30 sec intervals for 20 min (left ventricle samples) or 40 min (atrial samples). After exclusion of the first two minutes, in which the reaction was allowed to ‘settle’, fluorescence values were plotted against time. The slopes, representing the reaction rates (in ΔFluorescence × sec^−1^) were then extracted by linear regression and plotted for statistical analysis.

### ELISA for T-peptide binding to MMP-treated collagen IV

Black-wall, 96-well plates with transparent bottom (Optiplate; PerkinElmer) were coated with human (Merck, Australia) or mouse collagen IV (Cultrex, R&D Systems, MN, USA). Both collagens were diluted in 0.1 M acetic acid to achieve a final coverage of 20 µg per cm² (132 µg/ml; 50 µl per well). The plates were placed in an oven at 39 °C for 4 h to fully dry the collagen solution, then washed once with 100 µl/well of distilled water. Wells were then incubated overnight in a humidified chamber at 37 °C, in quadruplicates, with 80 µl/well of the following solutions: (A) DMEM alone (control), or (B) MMP-rich, serum-free conditioned medium from phorbol 12-myristate 13-acetate (PMA)-stimulated (50 nM; 24 h) HT-1080 human fibrosarcoma cells. Wells were subsequently washed 3 times with TBS (50 mM Tris, 150 mM NaCl, pH 7.4) and incubated with a blocking solution (TBS with 1% BSA, pH 8.0) for 1 h at room temperature, then washed again 3 times with TBS. Fluorescein isothiocyanate (FITC)-conjugated T-peptide (10 µg/ml, in TBS) was next added to each well for 1 h at room temperature. After another single TBS wash, FITC fluorescence (Ex/Em 483/530 nm) was measured on the CLARIOstar Plus multi-well plate reader (BMG Labtech, Australia). Background fluorescence from a well without collagen (incubated with DMEM) was finally subtracted and the mean fluorescence intensity of each group was determined.

### Statistical analysis

Statistical analyses were performed using GraphPad Prism version 10.4.1. Data sets were tested for normality by the Shapiro–Wilk normality test and presented as mean ± SD, or standard error of the mean (SEM), as specified in the figure legends. Comparison of grouped datasets was performed by two-way analysis of variance (ANOVA), or repeated-measures two-way ANOVA, to examine the main effect of the genotype, organ, tracer and their interaction on tracer uptake. Specific differences between two groups were then discerned by Sidak’s post-hoc analysis corrected for multiple comparisons. Standalone pairwise analyses employed a two-tailed Student’s *t* test. A *p*-value < 0.05 was considered significant.

## Supplementary information

Supplementary Information

Supplementary movie1

## Data Availability

The data that support the findings of this study are either provided within the article, or available from the corresponding author upon a reasonable request. The complete raw and normalized mRNA microarray data are available through the Gene Expression Omnibus of the National Centre for Biotechnology Information (http://www.ncbi.nlm.nih.gov/geo/, accession number GSE12420).
