# Supplementary material for: Collagen-targeted tracers for molecular imaging of atrial fibrosis and sensitive detection of atrial fibrillation
Source: NPJ Cardiovasc Health. 2025 Oct 6;2:49. doi: 10.1038/s44325-025-00086-2 (PMC12912425; doi:10.1038/s44325-025-00086-2)
Supplement: Supplementary file 1 — Supplementary Information [file 44325_2025_86_MOESM1_ESM.pdf]

Supplementary Information

Niego *et al*., Collagen-targeted tracers for molecular imaging of atrial fibrosis and sensitive detection of atrial fibrillation

Supplementary Figure 1. Pharmacokinetics and plasma stability of EP-3533 and the T-peptide collagen-targeted probes in the mouse. (a) A representative near-infrared scan of blood samples (diluted 1:20 in PBS) at 2, 12 and 240 min-post intravenous (*IV*) injection of Cy5.5-conjugated EP-3533 (0.5 mg/kg) to a double transgenic 'AF+HF' mouse (30 weeks). **(b)** Fitting of the clearance curves to a one-phase exponential decay model yields an EP-3533 blood half-life of 11.1±2.3 min. Mean±SEM, n=4 (3 males + 1 female; 3 transgenic + 1 control). **(c)** Only 4.7% of the tracer remains in the blood at 4 h post-*IV* administration (when the perfused heart and organ scans were performed; Figure 3). Mean±SD, n=10 (4 males + 6 females; 7 transgenic + 3 controls). ****$p<0.0001$ by a student t-test. **(d)** A blood clearance curve of Cy5.5-conjugated T-peptide (0.5 mg/kg) following *IV* injection of the tracer to double transgenic 'AF+HF' mice (30 weeks). The T-peptide blood half-life is 9.23±0.9 min. Mean±SEM, n=8 (3 males + 5 females; 3 transgenic + 5 controls). **(e)** High-performance liquid chromatography (HPLC) traces of Cy5.5-labeled T-peptide in citrated plasma of naïve C57Bl/6 mouse after incubation for 5, 10, 30, 60 and 240 min at 37°C. A blank trace of mouse plasma and T-peptide without plasma serve as controls. 60% of the T-peptide remains intact in mouse plasma at 60 min, but only 22% by 4 h.

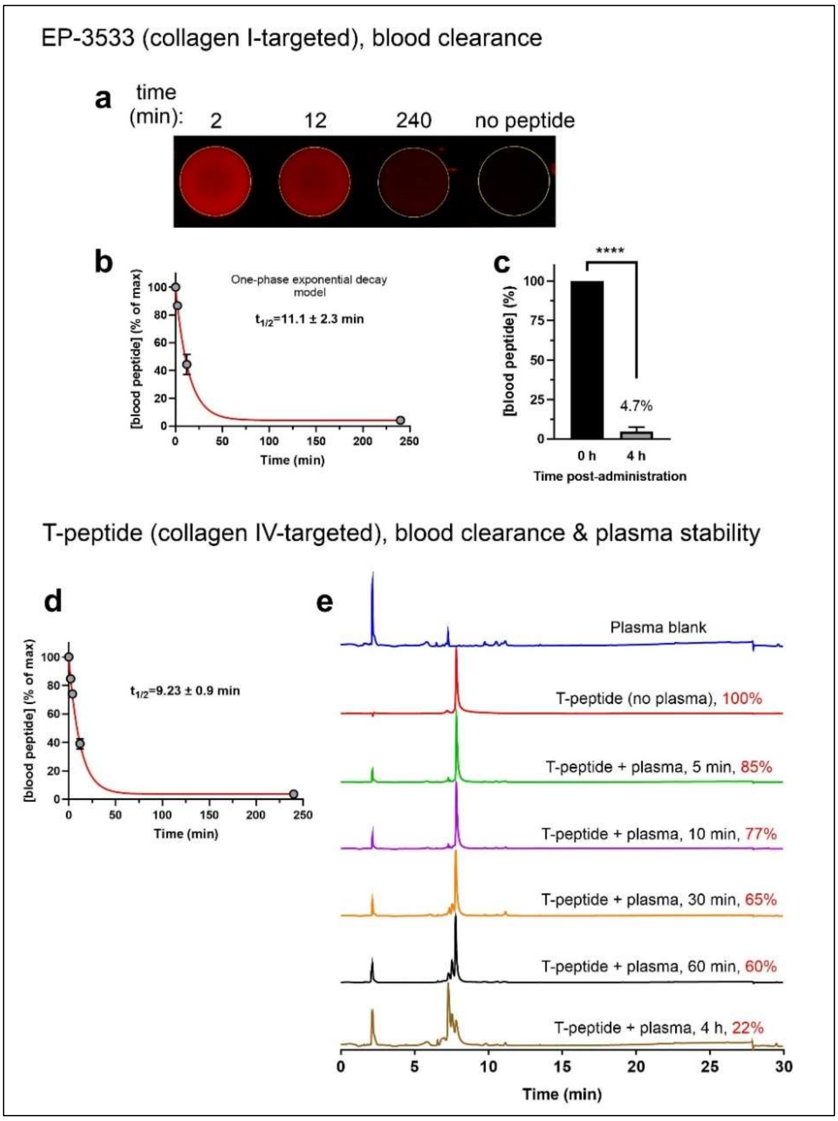

Supplementary Figure 2. Standard curves depicting a 2.22-fold higher brightness of Cy5.5-S-peptide (mutated peptide control) relative to the Cy5.5-T-peptide tracer on a mole-per-mole basis.

Cy5.5-T- and S-peptide Stardard Curves

	Mean Cy5.5 per pixel		
nM	Cy5.5-T-peptide	Cy5.5-S-peptide	Ratio S/T
130	231.75	502.62	2.17
13	17.03	36.58	2.15
1.3	1.63	4.22	2.58
0	0	0	

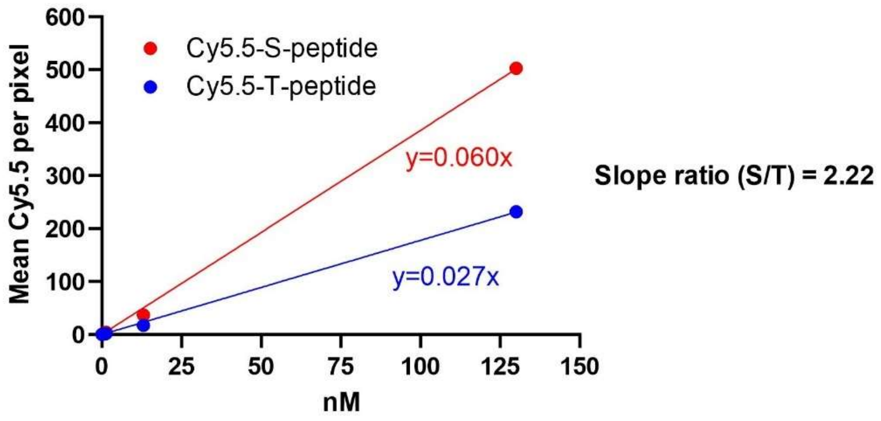

Supplementary Figure 3. Enhanced matrix metalloproteinase (MMP) activity around the fibrotic atrial endocardium in 'AF+HF' mice may increase the binding capacity of collagen IV to T-peptide. **(a)** In situ zymography using DQ™-gelatin showing large atrial patches with increased MMP activity (green) in direct contact with T-peptide-decorated fibrotic atrial endocardium (purple) in 'AF+HF' transgenic mice (lower panels). In contrast, atria of non-transgenic control mice (upper panels) possess much smaller and sporadic areas with MMP activity that do not localise with the atrial endocardium. Cell nuclei are depicted in blue (Hoechst). Tracer labeling was performed *in vivo* by intravenous injection of Cy5.5-conjugated T-peptide (0.5 mg/kg; 4 h), followed by *in situ* zymography *ex vivo*. The right panels are magnifications of the yellow squares in the middle panels. Images are from two separate mice per genotype (total mice tested: n=3 for control and n=4 for 'AF+HF'). Scale bars = 100 µm in the left and middle panels, 20 µm in the right panel. **(b, c)** The binding of Fluorescein isothiocyanate (FITC)-conjugated-T-peptide to human **(b)** and mouse **(c)** collagen IV is increased after collagen treatment with MMP-rich, serum free conditioned medium from PMA-stimulated (50 nM, 24 h) HT1080 human fibrosarcoma cells. The MMP content in the medium is demonstrated by gelatin zymogram in **(d)**. Mean±SD and two-tailed student t-test in (b), Mean in (c). n=3 for human collagen, n=2 for mouse collagen.

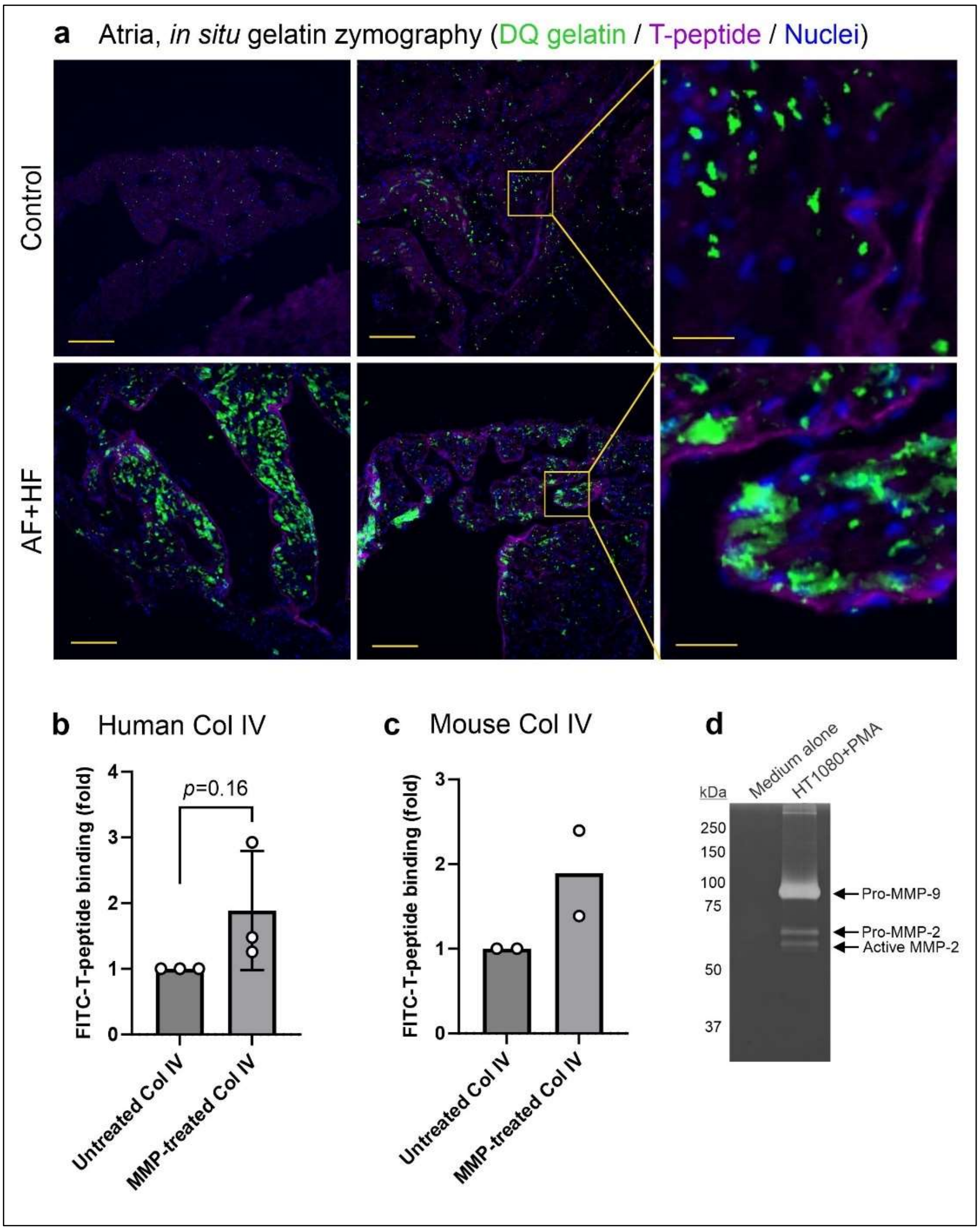

Supplementary Figure 4. Estimation of compartment-specific cardiac tracer uptake *in vivo* by T-peptide-based PET imaging (with no motion correction) demonstrates enhancement of the fibrotic atria in AF+HF mouse. (a) Representative coronal CT image of an 'AF+HF' transgenic mouse heart (left) and the corresponding single-bed PET/CT image (right) showing cumulative cardiac accumulation of ^{64}Cu-T-peptide-MeCOSar (0.25 mg/kg; 25.86±2.16 MBq) between 30-45 min post-tracer administration. The whole heart border is encircled by a yellow dashed line on the upper panels, while the estimated boundaries of the atria and ventricles are delineated on the lower panel in yellow and red dashed lines, respectively. **(b)** Quantification of the cumulative PET signal in each heart compartment (in kBq/cc; acquisition parameters as in (a)) comparing 'AF+HF' mice to non-transgenic littermate controls (28-30 weeks). Strong enhancement of the transgenic (fibrotic) atria is observed relative to controls. Mean±SD. n=4 for control and n=5 for 'AF+HF' (male mice in red, female mice in black or grey). *p<0.05, **p<0.01 and specified p-values by repeated-measure 2-way ANOVA with Sidak's post-hoc. Scale bar as indicated on the image, CT – computed tomography, kBq/cc - kilobecquerel per millilitre, MeCOSar - 5-(8-methyl-3,6,10,13,16,19-hexaaza-bicyclo[6.6.6]icosan-1-ylamino)-5-oxopentanoic acid, PET – positron emission tomography, CT – computed tomography, kBq/cc - kilobecquerel per millilitre, LA – left atrium, PET – positron emission tomography, RA – right atrium, V - ventricles.

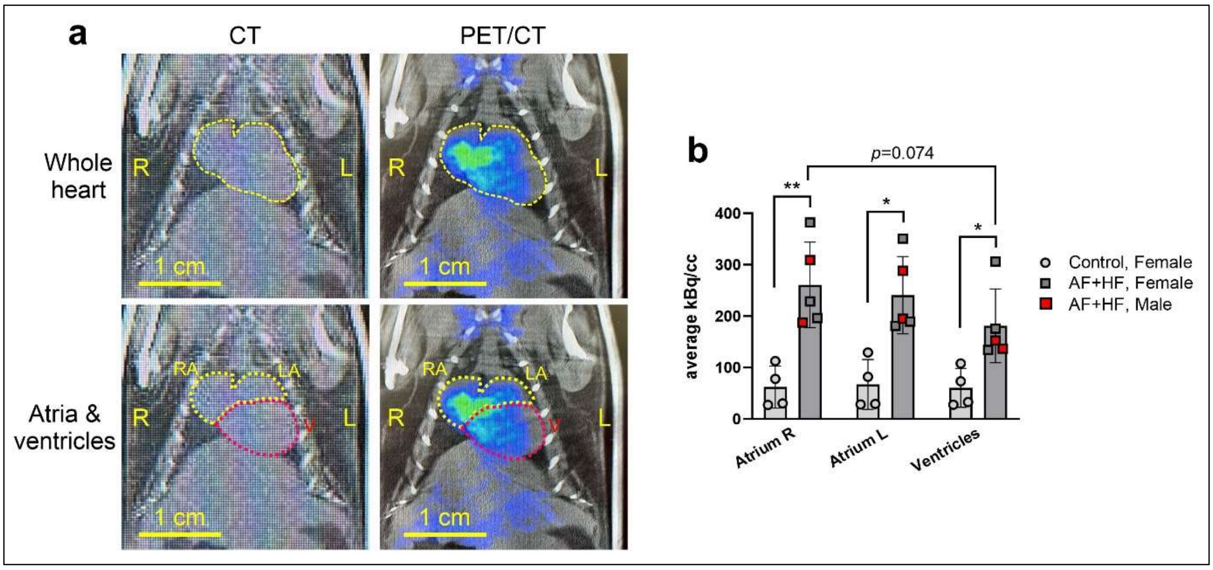

Supplementary Movie 1. Co-staining of fibrotic atrial tissue for T-peptide and intact collagen IV demonstrates T-peptide binding in close proximity to, but distinct separation from intact collagen.

A movie depicting 3d reconstruction from Z-stack of co-stained tissue section from the fibrotic atria of 'AF+HF' transgenic mouse. Tracer labeling was performed *in vivo* by intravenous injection of Cy5.5-conjugated T-peptide (0.5 mg/kg; 4 h; red), followed by collagen IV immunohistochemistry *ex vivo* (green). Cell nuclei are depicted in blue (Hoechst). The tracer binds the fibrous atrial muscle endocardium in close contact with, but district separation from intact collagen IV.
